# Detection of *Helicobacter pylori* and the Genotypes of Resistance to Clarithromycin, Fluoroquinolones, and Metronidazole in Gastric Biopsies: An In Silico Analysis to Help Understand Antibiotic Resistance

**DOI:** 10.3390/cimb47030187

**Published:** 2025-03-13

**Authors:** Pedro Valada, Ana Mata, Rui M. M. Brito, Teresa Gonçalves, José A. Medeiros, Célia Nogueira

**Affiliations:** 1 CNC-UC—Center for Neurosciences of Coimbra, University of Coimbra, 3000-504 Coimbra, Portugal; pedrovalada91@gmail.com (P.V.); tgoncalves@fmed.uc.pt (T.G.); 2 CiBB—Centre for Innovative Biomedicine and Biotechnology, University of Coimbra, 3000-504 Coimbra, Portugal; 3 Faculty of Medicine, University of Coimbra, 3000-504 Coimbra, Portugal; 4 Coimbra Chemistry Center, Institute of Molecular Sciences (CQC IMS), Department of Chemistry, University of Coimbra, 3004-535 Coimbra, Portugal; ana.mata@student.uc.pt (A.M.); brito@ci.uc.pt (R.M.M.B.); 5 BSIM Therapeutics, Instituto Pedro Nunes, 3030-199 Coimbra, Portugal; 6 Gastroenterology Clinic, 3000-098 Coimbra, Portugal; medeiros@ci.uc.pt

**Keywords:** *Helicobacter pylori*, resistance, clarithromycin, fluoroquinolones, metronidazole, in silico analysis

## Abstract

Antibiotic resistance in *Helicobacter pylori* is increasing rapidly and emerging as a major factor in treatment failure. We aimed to identify genetic mutations associated with resistance to clarithromycin (23S rRNA peptidyl transferase), fluoroquinolones (*gyrA*), and metronidazole (*rdxA*), and to explore their mechanisms of action through molecular modeling. *H. pylori* detection and the molecular characterization of genes were conducted directly on gastric biopsies by real-time PCR followed by nucleotide sequencing. A 3D model was used to evaluate molecular interactions between the antibiotics and respective target proteins. *H. pylori* was identified in 66.7% of 33 patients. An analysis of *23SrRNA* revealed novel mutations that, by in silico analysis, do not appear to contribute to clarithromycin resistance. In *gyrA*, mutations in amino acid residues 87 and 91 had an incidence of 27%, and the in silico analysis revealed that these positions are relevant in the binding and resistance to fluoroquinolones. It is also reported for other mutations, some of which are never described. All *rdxA* mutations were missense, with R16H, M56V, H97T, G98S, A118T, V123T, and R131K predicted by in silico analysis to impact metronidazole resistance. Monitoring *H. pylori* gene mutations is crucial for tailoring effective antibiotic therapies. Our study advances personalized medicine by introducing novel methods to detect resistance-related mutations and uncovering the molecular mechanisms driving this resistance.

## 1. Introduction

*Helicobacter pylori* is characterized as a gram-negative bacterium, microaerophilic, spiral-shaped, and flagellated [1,2]. Currently, *H. pylori* infection is one of the most common chronic infections in humans, affecting approximately half of the world’s population [3,4,5]. The prevalence of the infection varies due to several factors, such as geographic area, age, ethnicity, and socioeconomic status [4,6]. Regarding the geographic area, developing countries show the highest levels, exceeding 80% in some regions of the globe [7]. Portugal has a high prevalence of *H. pylori* infection; among children, it varies between 31.6% and 66.25%, and in adults, it is between 60% and 85% [8,9]. Although most *H. pylori* infections are asymptomatic, they predispose to the development of several conditions such as gastric chronic inflammation, non-ulcer dyspepsia, gastric and duodenal ulcers, gastric cancer, and gastric mucosa-associated lymphoid tissue lymphoma (MALT) [3,4,5,10]. Currently, there are different options for treatment that include a proton pump inhibitor (PPI) that is associated with at least two of the following antibiotics: clarithromycin (CLR), metronidazole (MTZ), amoxicillin (AMX), tetracycline (TET), and levofloxacin (LVX) [5,8,11,12]. Over the years, an overall decrease in the efficiency of treatment has been observed [5], mainly due to bacterial resistance to antibiotics [3,5,11,12]. Primary resistance to clarithromycin ≥20%, fluoroquinolones ≥20%, metronidazole ≥76%, amoxicillin ≥30%, and tetracycline ≥15% was reported in several studies in developed countries [13,14]. In Portugal, a meta-analysis on *H. pylori* resistance indicated the following overall resistance rates: clarithromycin 42%, metronidazole 25%, ciprofloxacin 9%, levofloxacin 18%, tetracycline 0.2%, and amoxicillin 0.1% [8].

*H. pylori* acquires antibiotic resistance mainly through chromosomal mutations and not through horizontal gene transmission [12,15]. Even though clarithromycin is one of the most common antibiotics used in the treatment due to the acid stability, there are several punctual mutations present in the coding region of the *23S rRNA* peptidyl transferase gene, A2142C, A2142G, and A2143G [15,16,17,18], which decrease clarithromycin affinity to the ribosomes of the bacterium, leading to *H. pylori* resistance [16,19]. The mechanism of resistance to fluoroquinolones is based on point mutations in the *gyrA* and *gyrB* genes, which encode the enzyme DNA gyrase, the target of this group of antibiotics [12,15]. Several mutations have been described that occur in amino acids 86, 87, 88, and 91. The most frequent mutations occur in amino acids 87 (N87K) and 91 (D91N, D91G, D91Y) [12,13,15,19] in the region of DNA binding to the N-terminal domain of the *gyrA* gene, blocking the binding site to fluoroquinolones [20]. Metronidazole is a component of hybrid, concomitant, quadruple, and sequential therapies for *H. pylori* infection [3,5,11,21]. The activation of metronidazole produces cytotoxic radicals that induce the breakdown of the double DNA chain, destabilize the α-helix, promote the unwinding of the DNA, and ultimately cause cell death [21,22,23]. Mutations in the *rdxA* gene, which encodes an oxygen-insensitive NADPH nitroreductase, and in the *frxA* gene, which encodes the NADPH flavin oxidoreductase, have been identified as the main cause of metronidazole resistance. Missense, frameshift, and nonsense mutations in the *rdxA* and *frxA* genes are the most frequent [12,21,22,23].

In Portugal, antibiotic susceptibility testing for *H. pylori* is not routinely performed, so the knowledge of regional antimicrobial resistance is crucial to successful eradication. The aim of this study was to identify genetic mutations associated with clarithromycin, fluoroquinolones, and metronidazole resistance directly on gastric biopsy specimens and validate them by an in silico model of analysis.

## 2. Materials and Methods

### 2.1. Study Population

The present study was approved by the Ethics Committee of the Faculty of Medicine of the University of Coimbra (CE-089/2017), and informed consent was obtained from all subjects. Thirty-three symptomatic patients who presented for endoscopy at a private gastroenterology clinic at Coimbra were enrolled in the study. Four gastric biopsies (two from the antrum and two from the corpus) were taken from each patient during the endoscopy procedure and were immediately stored in liquid nitrogen and then transferred to −80 °C until further use.

### 2.2. DNA Extraction

The gastric biopsies were disrupted using the MagNaLyser (Roche^®^, Mannheim, Germany) and treated with Bacteria Lysis Buffer (Roche^®^, Mannheim, Germany) and Proteinase K (Qiagen, Hilden, Germany) for 10 min at 65 °C. The gastric biopsy DNA was extracted and purified with the MagNA Pure Compact equipment (Roche^®^, Mannheim, Germany) using the commercial MagNA Pure Compact Nucleic Acid Isolation Kit I (Roche^®^, Mannheim, Germany), according to the manufacturer’s instructions. DNA concentration (A260) and purity (A260/A280) were determined spectrophotometrically (NanoDrop, Thermo Fisher Scientific, Waltham, MA, USA). DNA was stored at −20 °C for further use.

### 2.3. Helicobacter pylori Detection

*H. pylori* detection was performed with real-time PCR in a LightCycler 2.0 Thermocycler (Roche^®^, Mannheim, Germany) using the FastStart SYBR Green Master kit (Roche^®^, Mannheim, Germany) and specific primers for the identification of the bacterial flagellar gene, as described by Podzorski R. et al. [24].

### 2.4. Molecular Characterization of Genes Encoding Antibiotic Resistance

Mutations in the genes that confer resistance to antimicrobials were analyzed, including *23S rRNA* peptidyl transferase for clarithromycin, *gyrA* for fluoroquinolones, and *rdxA* for metronidazole, using real-time PCR. PCR reactions were performed in the LightCycler 2.0 Thermocycler (Roche^®^, Mannheim, Germany) in a final volume of 20 µL using the FastStart SYBR Green Master kit (Roche^®^, Mannheim, Germany) and specific primers described elsewhere [25]. PCR products were verified by agarose gel electrophoresis followed by sequencing elsewhere. The obtained sequences were analyzed using the Basic Local Alignment Search Tool (BLAST; http://blast.ncbi.nlm.nih.gov/Blast.cgi (accessed on 23 April 2018)) and aligned with the *H. pylori* 26695 reference strain (access number GenBank AE 000511.1 GI: 6626253). The comparative analysis of all the sequences obtained for each gene with the *H. pylori* 26695 reference strain was performed using the BioEdit program (https://bioedit.software.informer.com/ (accessed on 30 April 2018)).

### 2.5. In Silico Mutational Analysis

23S rRNA Modeling and Analysis

The full gene sequence of subunit *23S* ribosomal RNA (*23S* rRNA) from *H. pylori* 26695 is available at GenBank [26] entry HPrrnA23S. BLASTN [27,28] was used to align the amplified PCR sequence to the *Escherichia coli 23S* rRNA available at GenBank entry J01695, region 3250 6153 (Figure S1. BLASTN was also used to align the gene fragments from the PCR amplicon and the samples (Table S1) to HPrrnA23S. The PDB [29] entry 1J5A was used to analyze the interaction between clarithromycin and the peptidyl transferase center within *23S* rRNA.

DNA Gyrase Subunit A Homology Modeling

The amino acid sequence of DNA gyrase subunit A (GyrA) from *H. pylori* (strain 26695) was obtained from the UniProt knowledgebase [30], entry P48370.

Using the BLAST utility in the Protein Data Bank (PDB) [27,29], PDB entry 4Z2C for DNA gyrase (subunits A and B) from *Streptococcus pneumoniae* (strain R6) was chosen as the template. This sequence has 56% identity with the query sequence, 76% positives, and no gaps. The X-ray three-dimensional structure 4Z2C has been determined at 3.19 Å resolution, with the protein in a complex with moxifloxacin. Its sequence is also found at the UniProt knowledgebase entry Q8DPM2. The HOGENOM6 database [31] and Gene Context Tool NG [32] were used to assess and support homology between DNA GyrA from both organisms (Figure S2). Multiple sequence alignment was performed using Clustal Omega (1.2.4) [33,34] for UniProt entry P48370 and PDB entries: 4Z2C, 5BS8, 5BTA, and 5CDQ (in complex with moxifloxacin); 4Z2D, 5BTG, and 5BTI (in complex with levofloxacin); 2XCT and 5BTC (in complex with ciprofloxacin); and 5BTD and 5BTF (in complex with gatifloxacin). Modeller [35] version 9.19 was used for the homology modeling of the UniProt entry P48370 amino acid sequence and also the sequences of our samples (Table S2). Visualization and image generation were conducted using UCSF Chimera 1.12.

Oxygen-Insensitive NADPH Nitroreductase

The amino acid sequence of oxygen-insensitive NADPH nitroreductase (RdxA) from *H. pylori* (strain 26695) is available at the UniProt knowledgebase [30] entry O25608.

Using the BLAST utility from the Protein Data Bank (PDB) [27,29], the chosen template for RdxA was PDB entry 3QDL with 100% sequence identity with the query sequence of 210 amino acids. Structure 3QDL has been determined at 2.00 Å X-ray resolution and is complexed with flavin mononucleotide (FMN), with two functional homodimers present in the asymmetrical crystallographic unit [36].

As PDB entry 3QDL had a 100% identity for the sequence but had missing residues in the model from positions 97 to 128, an existing model was chosen from the AlphaFold repository [37,38]. Using UCSF Chimera 1.14 [39], it superimposed chains A and B of the homodimer from 3QDL. Modeller [35] version 9.19 was used for modeling the mutations on the RdxA model, exclusively on the position. Amino acid sequences for RdxA and the samples can be found in Table S3. UCSF Chimera 1.14 [39] was used to eliminate all ligands and water molecules, except for FMN and water molecules involved in H-bonds with this cofactor [36]. Visualization and image generation were conducted with UCSF Chimera 1.14 [39].

## 3. Results

### 3.1. Molecular Analysis of Genes Involved in H. pylori Antibiotic Resistance

Of the 33 patients enrolled in this study, 22 (66.7%) were positive for *H. pylori*. The 22 positive samples were analyzed to detect specific mutations in the *23S rRNA*, *gyrA*, and *rdxA* genes by a PCR-based sequencing approach. The analysis of the V domain of *23S rRNA* revealed several point mutations at the positions 2006 (A2006T, in 6% of the specimens), 2186 (T2186C, in 12%), 2206 (T2206A, in 12%), 2227 (A2227G, in 6%), and 2241 (T2241G, in 6%). Regarding fluoroquinolones, the quinolone resistance determining region (QRDR) of GyrA (nucleotide 142-606) was amplified and sequenced, allowing the identification of the most well-known point mutations in the 87 and 91 amino acids. In 20% of the specimens, there was an amino acid substitution at Asn-87 (N87T/I/K), and 7% had an amino acid substitution at Asp-91 (D91Y). Other mutations were found with a high prevalence, namely, mutations in codons 191 (M191I, in 93% of the isolates), 199 (V199A, in 60%), and 208 (G208E, in 87%). Furthermore, new mutations were detected for the first time, specifically at positions 145 (D145E, in 7%), 200 (H200Y, in 7%), and 219 (P219A, in 7%). Concerning metronidazole, we observed several point mutations that were translated into different amino acid substitutions, such as those in positions 6 (Q6H), 16 (R16H), 30 (S30R), 56 (M56V), 59 (D59N), 90 (R90K), 97 (H97T), 98 (G98S), 106 (P106L), 108 (S108A), 118 (A118T), 123 (V123T), 131 (R131K), 172 (V172I), and 183 (A183V). The mutation at amino acid 59 was the most prevalent, found in 86% of the cases, followed by mutations at amino acid 6 (71%) and at amino acid 90 (57%); amino acid modifications in positions 98, 118, and 131 occur at a representative frequency of 43%. A new variant emerges from this study, namely, V123T (14%).

### 3.2. In Silico Mutational Analysis

#### 3.2.1. 23S rRNA

**Figure 1:**
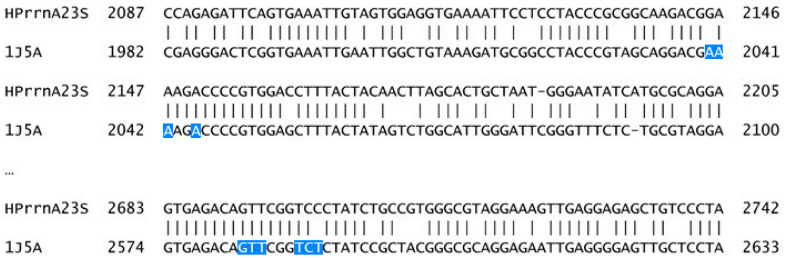
Alignment of HPrrnA23S with a gene fragment present in PDB entry 1J5A. The nucleotides boxed in blue are known to interact with clarithromycin.

Versalovic et al. [40] concluded that a single-point mutation within domain V (peptidyl transferase loop) of *23S* rRNA from *H. pylori* might be enough to induce resistance to macrolides such as clarithromycin. The most commonly described single-point mutations in the DNA sequence that fit that criterion, A2058G and A2059G (*E. coli* numbering scheme) [40,41], are not within—nor near—the samples sequenced in this work (the alignment is shown in Figure S1). The only structure available in the PDB that has clarithromycin complexed in the peptidyl transferase center (Figure 1) showed that clarithromycin interacts with nucleotides A2146 and A2147, as well as some others near them, which are locations within the range of our samples.

#### 3.2.2. DNA Gyrase Subunit A Homology Modeling

**Figure 2:**
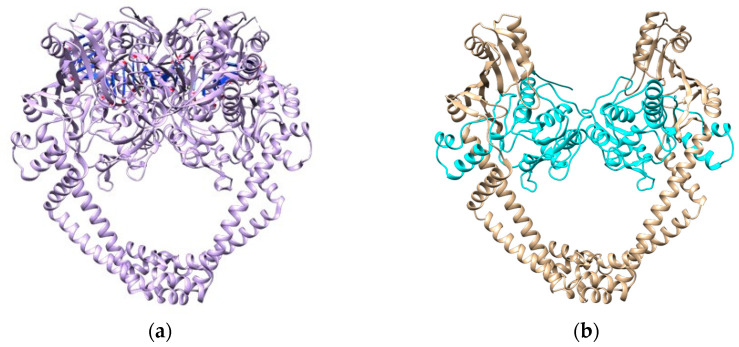
Schematic representations of the tridimensional structures of DNA gyrase: (**a**) PDB entry 4Z2C used as a template and (**b**) the homology model of P48370 DNA GyrA. In (**b**), the cyan chain represents amino acids 1 to 220 (the last position covered by the samples sequenced in this work).

In the absence of a three-dimensional (3D) structure for the P48370 sequence, a model of the 3D structure of DNA GyrA from *H. pylori* (strain 26695) was obtained by homology modeling. Out of 37 PDB entries with at least 50% identity to the FASTA sequence, 14 were complexed with fluoroquinolones. PDB entry 4Z2C was the best ranked and was used as a template for the homology modeling after verifying the sequence homology of DNA gyrase subunit A amongst the different organisms (Figure 2).

**Figure 3:**
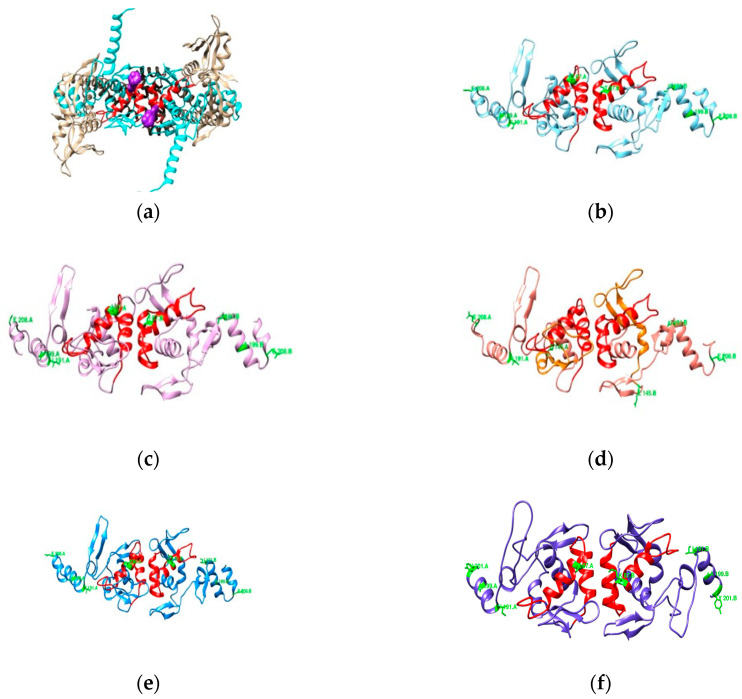
Top view of several structural models created for different sequences and respective QRDRs (amino acids 71–110 in red). (**a**) Homology model of the P48370 sequence with moxifloxacin (purple) transferred from the template structure 4Z2C. (**b**) 04, (**c**) 06, and (**d**) 09, with part of the CAP domain in orange (111–145). (**e**) 15 and (**f**) 20 are structural models for each respective sequence, with mutations represented in green.

To ascertain possible resistance to fluoroquinolones, we looked into the quinolone resistance determining region (QRDR, Figure S2) [42]. According to Higgins et al. [42], significant homology exists in the QRDR and in the CAP domain of eubacterial DNA gyrases, amino acids 70–141 (numbered according to the *E. coli* sequence), which is also where resistance mutations occur. Aligning our sequence P48370 (*H. pylori* 26695) to the sequence from the UniProt entry P0AES4 (*E. coli* strain K12), this range of the CAP domain translates to positions 74–145, with the QRDR ranging from amino acids 67 to 106 (tr. 71 to 110 in *H. pylori*) and the most relevant positions for resistance mutations at 83 and 87 (tr. 87 and 91 in *H. pylori*) [42,43]. From our samples, only sequences 04, 06, 15, and 20 have mutations in the QRDR (Figure 3b,c,e,f), which prompted further analysis. Even though sample 09 has mutations outside the QRDR, one is included at the end of the CAP domain (D145E) and, as such, was also analyzed (Figure 3d).

The proximity of this binding site to the QRDR, above the α-helices, shows the importance of mutations in positions 87 and 91 in conferring resistance to this class of antibiotics [42,43]. Further alignments of P48370 with structures from the PDB that are complexed with fluoroquinolones (Figure S3) indicated that positions 87 and 125 of P48370 (*H. pylori* 26695) were the most relevant in the drug-binding interaction. Samples 04, 06, and 20 have a mutation at position 87 from asparagine to threonine, isoleucine, and lysine, respectively. While samples 09 and 15 have no mutations at this position, they have mutations inside the CAP domain (amino acids 74–145, as seen in Figure S2) that encompass both positions 87 and 125, especially sample 15 with a mutation at position 91 (near 87 and inside the QRDR) from an aspartate to a tyrosine.

#### 3.2.3. RdxA

**Figure 4:**
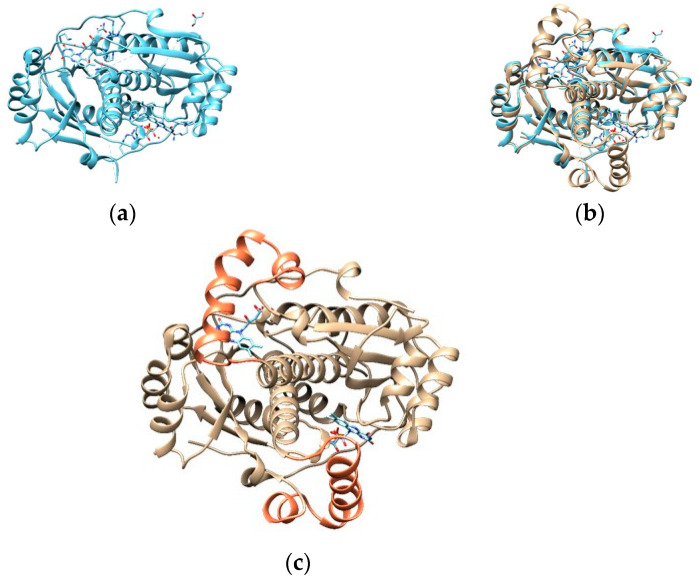
Structures of RdxA: (**a**) 3QDL PDB entry (blue); (**b**) superimposition of the AlphaFold model (tan) to each of the chains of 3QDL; and (**c**) final template used for sample modeling, with the missing residues highlighted in orange (FMN in blue).

With a considerable part of the sequence missing from the known crystallographic structure for RdxA (PDB entry 3QDL, shown in Figure 4a), a complete model for the UniProt entry O25608 was obtained from the AlphaFold repository [37,38]. After combining the chains and transferring the FMN ligands and selected waters [36] from 3QDL, we obtained a new RdxA model (Figure 4c) that was used henceforth.

**Figure 5:**
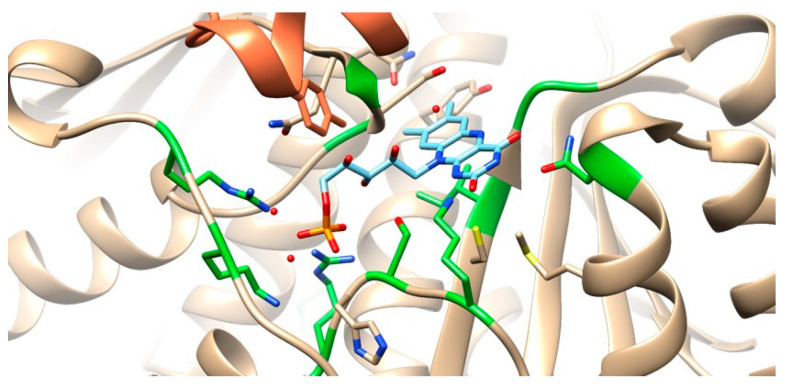
Close-up of the binding site of one of the FMNs (light blue) of the RdxA model.

Martínez Júlvez et al. [36] (where PDB entry 3QDL was presented) report some known regions where mutations might lead to a loss of function, as they are considered binding residues (represented in green in Figure 5): Arg16, Ser18, Lys20, Ser45, Tyr47, Asn73, Ile160, Gly162, Gly163, Lys198, and Arg200.

Binding residues mentioned by Martínez Júlvez et al. [4] are represented in green, with their side chain explicit.

From the analysis of our samples, the mutations in positions expected to cause a loss of function are R16H, M56V, P106L, and A183V [36,44].

**Figure 6:**
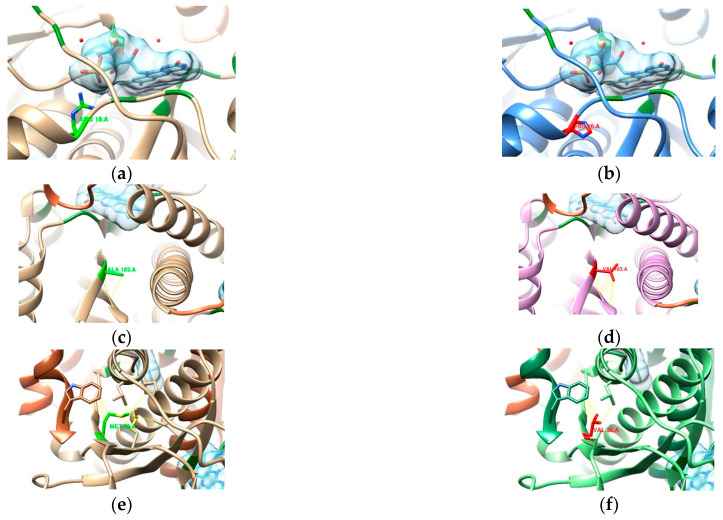

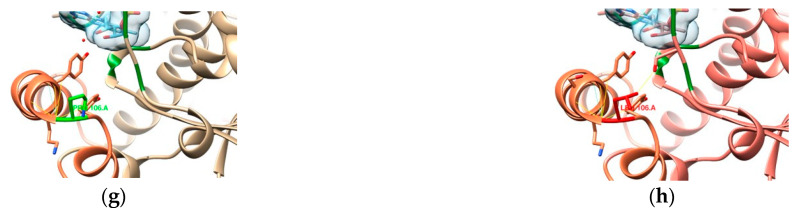
Comparison between the templates (**a**,**c**,**e**,**g**), highlighting the residue in light green, and the mutated models (mutated residues in red): (**b**) R16H, (**d**) A183V, (**f**) M56V, and (**h**) P106L. An FMN is represented with a surface in light blue and other binding residues in dark green. Blue and yellow lines represent expected H-bonds and close contacts, respectively. In (**e**,**f**), the other chain of the dimer is in a darker color.

Mutations in residues 16 and 183 are known to decrease affinity to oxidoreductases [36,44]. In the R16H model (Figure 6a,b), while similar in chemistry, His16 has a shorter side chain that shows the loss of possible H-bond interactions with FMNs. As for the A183V model (Figure 6c,d), the change in amino acids does not seem to affect any apparent interaction with the FMN. Any contribution to resistance might be related to a higher rigidity in the β-sheet—as the longer chain establishes more interactions—and that might affect the conformation of the binding site to accommodate the FMN.

In residue 56, mutations usually destabilize homodimer formation [36,44], and the reason for this can be observed in model M56V (Figure 6e,f), as the mutation happens at the interface of both chains. A sulfur-containing amino acid like methionine mutated to a shorter, aliphatic valine predicts the loss of four contacts (originally nine) that stabilize the structure at the interface (allowing for other contacts present in the dimer).

The last position that we know of that is connected with a loss of function of the reductase is at position 106, where—in our samples—a proline (aliphatic, forming a ring with the backbone) is mutated to a leucine (aliphatic). Model P106L (Figure 6g,h) shows that this position seems to stabilize the beginning of the α-helix where the original proline forms a total of seven contacts (two of which are H-bonds). With leucine, this number increases to nine contacts (maintaining the two H-bonds). Martínez Júlvez et al. [36] describe this region/helix as protected against proteolysis when bound to the NADP^+^ cofactor. It is found on a solvent-exposed interface near the FMN, and its mobility might be crucial for optimal binding and catalysis [36]. From what is observed in Figure 6g,h, the new contacts formed with the leucine variant are right at the beginning of the helix, so the mobility might not be too different from the consensus proline. Still, it is important to highlight that this position is found in the region of the missing residues from the PDB entry 3DQL, so this analysis is based solely on the theoretical model of the template obtained from AlphaFold.

**Figure 7:**
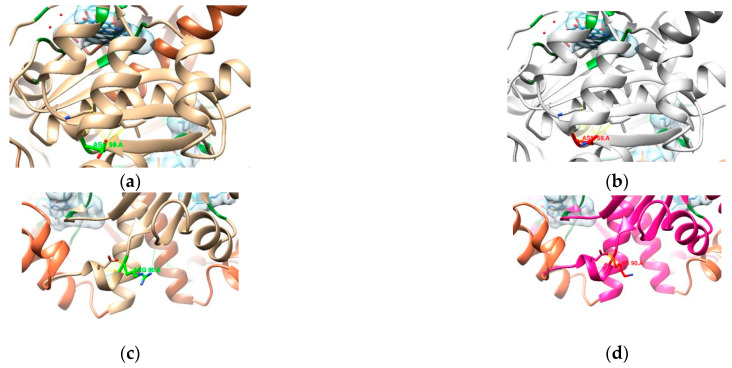
Comparison between the templates (**a**,**c**), highlighting the residue in light green, and the mutated models (mutated residues in red): (**b**) D59N and (**d**) R90K. An FMN is represented with a surface in light blue and other binding residues in dark green. Blue and yellow lines represent expected H-bonds and close contacts, respectively.

In the other samples, the mutations that occurred most were D59N, R90K, G98S, A118T, and R131K. All of these are common in metronidazole-resistant RdxA strains [44,45]. From our in silico analysis, these single-point mutations do not seem to be resistance-conferring by themselves but could be considered secondary/auxiliary mutations leading to resistance. While not close to the binding site, residue Asp59 (Figure 7a) seems to be responsible for maintaining the α-helix that interfaces with the FMN. Mutation D59N (acidic aspartate replaced by the amidic asparagine; Figure 7b) seems to create more spaced interactions, facilitating the destabilization of the “flap” in which the helix is involved and consequently destabilizing the position of the FMN inside the binding pocket.

In mutation R90K (Figure 7c,d), the amino acid chemistry and size are similar, but the loss of two amine groups affects the interactions involved in stabilizing a central part of the RdxA structure. It is possible that this mutation helps cause a loss of function.

**Figure 8:**
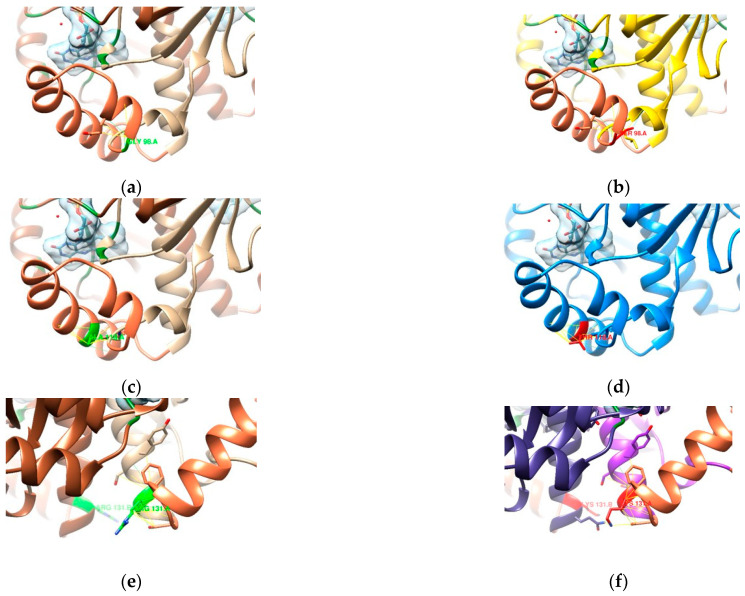
Comparison between the templates (**a**,**c**,**e**), highlighting the residue in light green, and the mutated models (mutated residues in red): (**b**) G98S, (**d**) A118T, and (**f**) R131K. An FMN is represented with a surface in light blue and other binding residues in dark green. Blue and yellow lines represent expected H-bonds and close contacts, respectively. In (**e**,**f**), the other chain of the dimer is in a darker color.

Mutation G98S (Figure 8a) happens inside the region of the model that contains the missing residues. As was mentioned above, the mobility of this region might be essential for optimal binding [36], and, as seen in Figure 8b, the presence of Ser98 will most certainly insert some degree of rigidity in the solvent-exposed interface near the FMN. Extra interactions with the small helix precedent to the missing residues will tighten the loop where position 98 is included. Similarly, the substitution of alanine (aliphatic; Figure 8c) to threonine (hydroxylic; Figure 8d) in A118T suggests the same reason for causing resistance: the presence of a hydroxyl group is predicted to form more interactions (six more than Ala118 in this model), creating rigidity in this region by compacting the helix.

The final most recurring mutation, R131K (Figure 8e,f), similar to R90K (Figure 7c,d), is a substitution between two basic residues, from arginine to lysine, with the loss of two amine groups, which can be involved in relevant interactions. The more extended conformation of Lys131—placed at the interface between the protomers—leads to a predicted increase in the number of contacts, including the region of missing residues and with the other protomer. The resulting more compact dimer might have the mobility of this region affected at its ending loop, accompanied by a more solvent-accessible interface with the FMN.

**Figure 9:**
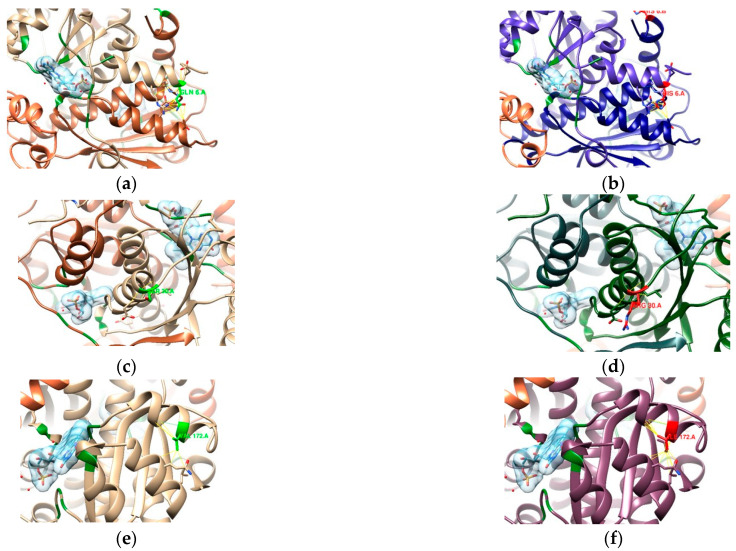
Comparison between the templates (**a**,**c**,**e**), highlighting the residue in light green, and the mutated models (mutated residues in red): (**b**) Q6H, (**d**) S30R, and (**f**) V172I. An FMN is represented with a surface in light blue and other binding residues in dark green. Blue and yellow lines represent expected H-bonds and close contacts, respectively. In (**a**–**d**), the other chain of the dimer is in a darker color.

**Figure 10:**
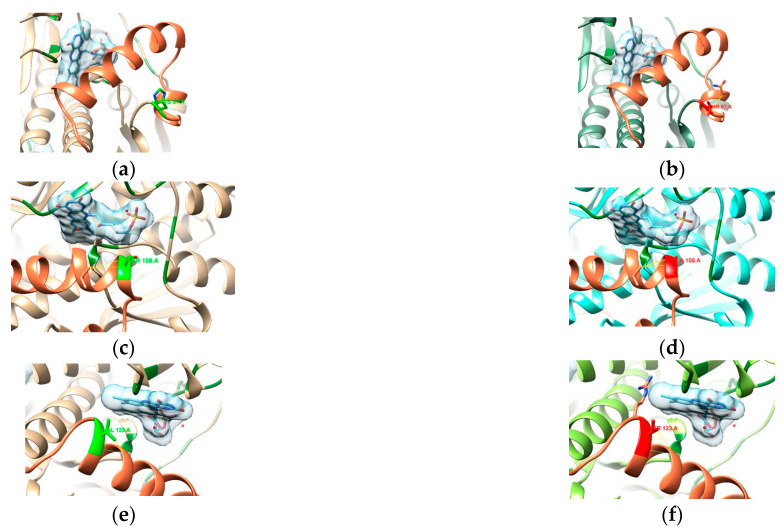
Comparison between the templates (**a**,**c**,**e**), highlighting the residue in light green, and the mutated models (mutated residues in red): (**b**) H97T, (**d**) S108A, and (**f**) V123T. An FMN is represented with a surface in light blue and other binding residues in dark green. Blue and yellow lines represent expected H-bonds and close contacts, respectively.

As for the remaining six missense mutations found in the samples, we divided their analysis into two sections: outside (Q6H, S30R, and V172I; Figure 9) and inside (H97T, S108A, and V123T; Figure 10) the region of missing residues. Overall, these mutations could also be considered auxiliary mutations leading to resistance to metronidazole.

Mutation Q6H causes the substitution of the amidic glutamine by the basic residue, histidine. The consensus Gln6 (Figure 9a) found at the interface of the protomers seems to be relevant in dimerization (19 contacts predicted, with five being interactions with the other protomer) that help define the structure of RdxA. With His6 (Figure 9b), the number of predicted contacts increases up to 33 (15 with the other protomer). The Q6H mutation might lead to a more compact dimer by interacting with the residues found in positions 31 and 35 of the other protomer (a helix followed by a loop at the interface with the FMN).

That region is where mutation S30R occurs. The replacement of Ser30 (hydroxylic; Figure 9c) by the larger Arg30 (basic; Figure 9d) might affect the establishment of the helix’s starting point—the same number of interactions between the same residues but with slightly shorter contacts. This mutation does not seem to influence the overall status of the RdxA structure or any binding interfaces.

In V172I, the positioning of the Val172 (Figure 9e) in the structure seems to be involved in keeping the position of a “flap” that interfaces with the FMN. The mutated Ile172 (Figure 9f) predicts 12 more interactions (a total of 19), most of which are between the same residues found in interactions with Val172, except for four new contacts with another residue within the same helix (position 173). This might lead the mutated structure to have a more rigid “flap”—but as it interfaces at the edge of the FMN, it is possible that there is not as much of an effect on the binding site as a single mutation.

As stated before, the region of the missing residues needs to retain its high mobility for optimal binding [36]. Considering the analysis was made on a model, the results presented herein are just indicators of how the mutations might affect the structure of RdxA and lead to resistance.

Mutation H97T happens right at the beginning of the missing residue region His97 (basic; Figure 10a), which only shows a single interaction with the loop, while mutated Thr97 (hydroxylic; Figure 10b) forms more contacts with the next loop, which might affect the shape of the region to shorten the helix.

The missense mutation S108A happens at the beginning of the helix that interfaces directly with the FMN binding pocket. The contacts involved with Ser108 (hydroxylic; Figure 10c) within the helix are not very different from those involved with Ala108 (aliphatic: Figure 10d), so it might not lead to resistance.

In mutation V123T, Val123 (aliphatic; Figure 10e) is predicted to establish only three contacts within the α-helix and is replaced by Thr123 (hydroxylic; Figure 10f), with three more predicted interactions. Despite these extra interactions still being within the helix, they involve two more residues. As for H97T, it might increase rigidity in the zone, but its position at the edge might not be very impactful.

## 4. Discussion

The resistance of *H. pylori* to antimicrobial agents is increasing and can result in treatment failure and inappropriate antibiotic usage. So, up-to-date knowledge about antibiotic resistance rates in each geographic region is crucial to guide the best option for treatment. The phenotypic methods based on cultured bacteria have been the most used to detect antibiotic resistance, but molecular methods are gaining ground. In Portugal, where routine antibiotic susceptibility testing for *H. pylori* is not standard practice, understanding local antimicrobial resistance patterns is crucial, especially given the high resistance to the most widely used antibiotics [8]. Clarithromycin is one of the most used antibiotics in treatment regimens for the eradication of *H. pylori*, but the spread of clarithromycin resistance has led to guideline modifications. Mutations in the peptidyl transferase-encoding region of *23S* rRNA are responsible for resistance to clarithromycin; the most frequently described are A2142C, A2142G, and A2143G [15,16,17,18]; however, there are still other mutations associated with resistance to clarithromycin that appear with less frequency, such as A2144T, T2717C, and C2694A [19,25,46]. In this study, none of these mutations were found; however, we have identified several novel mutations in positions 2006 (A2006T), 2186 (T2186C), 2206 (T2206A), 2227 (A2227G), and 2241 (T2241G). Their role in clarithromycin resistance was evaluated by an in silico model, which revealed that none of them were present in the peptidyl transferase loop/region, and as such, they are not expected to contribute to resistance against clarithromycin.

*H. pylori* resistance to fluoroquinolones is mainly due to mutations present in genes encoding DNA gyrase (*gyrA* and *gyrB* genes), particularly in the quinolone resistance determining region (QRDR) of the *gyrA* gene, such as those affecting amino acids 86, 87, 88, 91, and 97. Of these, mutations in positions 87 and 91 are the most impactful. It has been reported that amino acid substitution at position 87 is related to a high level of resistance, whereas amino acid substitution at position 91 may be linked to low-level resistance [21,47,48]. In our study, both of the mutations were detected at a global prevalence rate of 27%. Taking into account that these mutations might be translated into a resistance phenotype to fluoroquinolones, we find that our data are corroborated by Lopo et al. in a systematic review and meta-analysis from Portugal, which also shows an overall resistance to fluoroquinolones of 27% [8]. When this value is compared to data from other countries where resistance phenotypes have similarly been detected using molecular methods, our rate appears to be higher; in a population from southern Italy, the prevalence of primary levofloxacin resistance was 19.2% [18], in an eastern German region it was 13.7% [49], and in Malta, it was 21.4% [50]. Moreover, in addition to these mutations, others were detected in this study and with a higher prevalence, namely in positions 191, 199, and 208. The codon substitution V199A was poorly correlated with fluoroquinolone resistance [51], which can be explained by the fact that the substituted amino acid has no impact on the structure of the GyrA subunit since the substitution of a valine to an alanine does not change the polarity of the side chain. The presence of new mutations may be due to a variety of point mutations from the loci of the *gyrA* gene that, although not yet reported, may be a fundamental factor in perceiving the predominance of resistance to fluoroquinolones. In this study, we also identified three novel mutations in codons 145, 200, and 219, and their impact on the structure of the GyrA subunit was analyzed through an in silico model. From the alignment of DNA gyrA from *H. pylori* with several DNA gyrA structures complexed with fluoroquinolone, the relevance of position 87 in binding was highlighted. Samples 04, 06, and 20 have a mutation in this position (aa 87), which might indicate the potential of these samples to display resistance against fluoroquinolones.

Furthermore, considering the type of mutation observed, from the wild-type arginine, i.e., a polar/amidic amino acid, to the non-polar/aliphatic isoleucine of sample 06 and the positively charged lysine of sample 20, there is a larger alteration in the binding site environment than in the case of sample 04, which also mutates to a polar amino acid (lysine) even if it is hydroxylic. Sample 15 has a mutation inside the QRDR, and even if there were no interactions seen at position 91, in *E. coli*, it is known for resistance-conferring mutations [43], and given the proximity to the binding site, it might also affect the environment, taking into account the substitution from an acidic amino acid (aspartate) to a larger aromatic one (tyrosine). Sample 09 has a mutation at the end of the CAP domain, consisting of the substitution of an acidic amino acid (aspartate) by another acidic one (glutamate), which, combined with its position farther away from the binding site, suggests that this mutation would not affect the structure or environment enough to contribute to resistance against fluoroquinolones.

In the case of metronidazole, most of the described mutations are missense mutations, which are characterized by the substitution of an amino acid, when compared to the sequence of *H. pylori* 26695. In this study, missense mutations were found in six samples, and no frameshift or nonsense mutations were observed. Mutations at amino acids 16 and 183 are reported to be associated with decreased affinity for oxidonucleases [36,44], whereas the mutation at amino acid 56 has been correlated with the destabilization of dimer formation [36,44]. Taking into account our in silico analysis, we predict that mutations R16H, M56V, H97T, G98S, A118T, V123T, and R131K can lead to resistance. However, it is important to emphasize that the analysis of the H97T, G98S, A118T, and V123T mutations is based on a theoretical model without the corroboration of a crystallographic structure, which could provide greater clarity regarding whether they are primary resistance-conferring mutations or secondary mutations. Furthermore, most of them were found in both MTZ-resistant and MTZ-sensitive isolates [12,17,21,52,53,54,,55], with the exception of R16Q, which is mainly detected in resistant strains [17,21,44,47,54]. The in silico analysis of mutations Q6H, D59N, R90K, and V172I indicates that they do not appear to be resistance-conferring mutations, although they were observed in metronidazole-resistant strains, suggesting a role as auxiliary mutations. This assumption is corroborated in the literature since they are also detected in susceptible isolates [17,22,45,56]. As for mutations S30R, S108A, A183V, and P106L, they might not be resistance-conferring mutations by themselves due to the similarity between the original and mutated amino acids. In fact, most of the time, they are reported in MTZ-sensitive isolates [17,45,56,57,58]. Nevertheless, Butlop et al. found that the mutation at amino acid 106 is correlated with a loss of reductase function, thus promoting resistance to metronidazole [44].

This study has limitations that should be acknowledged. Firstly, the limited sample size; a larger sample size would be an added value to reinforce the strength of the data gathered. Secondly, it was a study conducted at a single medical center and may not be indicative of the population in a broader region. Lastly, the fact that culture and antibiotic susceptibility tests are not routinely carried out is a significant limitation, as it precluded the assessment of the phenotypic expression of the genetic mutations identified in this study.

## 5. Conclusions

In conclusion, the detection of mutations in *H. pylori* that confer antibiotic resistance is currently essential for effective treatment strategies since the occurrence of antibiotic resistance in *H. pylori* differs across countries and is influenced by the overall extent of antibiotic utilization. Our results show high levels of mutations conferring resistance to fluoroquinolones and metronidazole; however, the same was not observed for clarithromycin, as we did not find any resistance-conferring mutations.

This study introduces an innovative approach to detecting mutations associated with *H. pylori* resistance by integrating genomic analysis with an in silico model that simulates interactions between antibiotics and their mutated targets. The predictive value of these data allows for the optimization of treatments, enabling the refinement of personalized medicine.

## Data Availability

The original contributions presented in this study are included in the article/Supplementary Materials. Further inquiries can be directed to the corresponding author.

## Supplementary Materials

The following supporting information can be downloaded at: https://www.mdpi.com/article/10.3390/cimb47030187/s1.
