# Supplementary material for: Detection of Helicobacter pylori and the Genotypes of Resistance to Clarithromycin, Fluoroquinolones, and Metronidazole in Gastric Biopsies: An In Silico Analysis to Help Understand Antibiotic Resistance
Source: Curr Issues Mol Biol. 2025 Mar 13;47(3):187. doi: 10.3390/cimb47030187 (PMC11940985; doi:10.3390/cimb47030187)
Supplement: Supplementary file 1 [file cimb-47-00187-s001.zip › Supplementary files.pdf]

Table S1. Gene fragments of HPrrnA23S amplified by PCR and samples.

PCR amplification of HPrrnA 23S	CCACAGCGATGTGGTCTCAGCAAAGAGTCCCTCCCGACTGTTTACCAAAAACACAGCACTTT-<br>GCCAACTCGTAAGAGGAAGTATAAGGTGTGACGCCTGCCCGGTGCTCGAAGGTTAAGAGGATGCGTCAGTCGCAAGATGAAGCGTTGAATTGAAGCCCGAGTAAACGGCGGCCGTA<br>ACTATAACGGTCCTAAGGTAGCGAAATTCCTTGTCGGTTAAATACCGACCTG-<br>CATGAATGGCGTAACGAGATGGGAGCTGTCTCAACCAGAGATTCAGTGAAATTGTAGTGGAGGTGAAAATTCCTCCTACCCGCGGCAAGACGGAAAGACCCCGTGGACCTTTACTAC<br>AACTTAGCACTGCTAATGGGAATATCATGCGCAGGATAGGTGGGAGGCTTTGAAGTAAGGGCTTTGGCTCTTATGGAGCCATCCTTGAGATAC	*447071-447510*<br><br>1859-2225

Sample 13	CACTTTGCCAACTCGTAAGAGGAAGTATAAGGTGTGACGCCTGCCCGGTGCTCGAAGGTTAA-<br>GAGGATGCGTCAGTCGCAAGATGAAGCGTTGAATTGAAGCCCGAGTAAACGGCGGCCGTAACTATAACGGTCCTAAGGTAGCGAAATTCCTTGTCGGTTAAATACCGACCTGCATGA<br>ATGGCGTAACGAGATGGGAGCTGTCTCAACCAGAGATTCAGTGAAATTGTAGTG-<br>GAGGTGAAAATTCCTCCTACCCGCGGCAAGACGGAAAGACCCCGTGGACCTTTACTACAACTTAGCACTGCTAACGGGAATATCATGCGCAGGATAGGTGGGAGGCT	T2186C	*447127-447466*<br><br>1879-2218
Sample 17	GCCAACTCGTAAGAGGAAGTATAAGGTGTGACGCCTGCCCGGTGCTCGAAGGTTAAGAG-<br>GATGCGTCAGTCGCAAGATGAAGCGTTGAATTGAAGCCCGAGTAAACGGCGGCCGTAACTATTACGGTCCTAAGGTAGCGAAATTCCTTGTCGGTTAAATACCGACCTGCATGAATG<br>GCGTAACGAGATGGGAGCTGTCTCAACCAGAGATTCAGTGAAATTGTAGTG-<br>GAGGTGAAAATTCCTCCTACCCGCGGCAAGACGGAAAGACCCCGTGGACCTTTACTACAACTTAGCACTGCTAACGGGAATATCATGCGCAGGAAAGGTGGGAGGCTTTGAAGTAGG<br>GGCTTTGGCTCTGATGGAGAA	A2006T, T2186C, T2206A, A2227G, T2241A, C2248A, C2249A	*447133-447497*<br><br>1885-2249
Sample 23	GCACTTTGCCACTCGTAAGAGGAAGTATAAGGTGTGACGCCTGCCCGGTGCTCGAAGGTTAA-<br>GAGGATGCGTCAGTCGCAAGATGAAGCGTTGAATTGAAGCCCGAGTAAACGGCGGCCGTAACTATAACGGTCCTAAGGTAGCGAAATTCCTTGTCGGTTAAATACCGACCTGCATGA<br>ATGGCGTAACGAGATGGGAGCTGTCTCAACCAGAGATTCAGTGAAATTGTAGTG-<br>GAGGTGAAAATTCCTCCTACCCGCGGCAAGACGGAAAGACCCCGTGGACCTTTACTACAACTTAGCACTGCTAATGGGAATATCATGCGCAGGAAAGGTGGGAGGCTTTGAAGTAAG<br>GGCTTTGGCTCT	A1888del, T22	*447126-447135, 44713*

		06<br>A	*8-447*<br>*488*<br><br>1878-<br>1887,<br>1889-<br>2240

Table S2. Sequence of amino acids from P48370 and the samples studied.

P4<br>83<br>70	MQDNSVNETKNIVEVGIDSSIEESYLAYSMSVIIGRALPDARDGLKPVHRRILYAM-<br>HELGLTSKVAYKKSARIVGDVIGKYHPHGDNAVYDALVRMAQDFSMRLELVDGQGNFGSIDGDNAAAMRYTEARMTKASEEILRDIDKDTIDFVPNYDDTLKEPDILPSRLPNLLVNGANGIAVGMATSI<br>PPHRMDEIIDALVHVLENPNAGLDEILEFVKGPDFPTGGIIYGKAGIIEAYKT-<br>GRGRVKVRAKVHVEKTKNKEIIVLDEMPFQTNKAKLVEQISDLAREKQIEGISEVRDESDREGIRVVIELKRDAMSEIVLNHLYKLTTMETTFSIILLAIYNKEPKIFTLLELLHLFLNHRKTIIIRRTIFELE<br>KAKARAHILEGYLIALDNIDEIVRLIKTSQSPEAAKNALMERFTLSEIQSKAILEMRLQRL-<br>TGLERDKIKEEYQNLLELIDDLNGILKSEDRLNGVVKTELLEVKEQFSSPRRTEIQESYENIDIEDLIANEPMVVSMSYKGYVKRVDLKAYEKQNRGGKGKLSGSTYEDDFIENFFVANTHDILLFITNKGQ<br>LYHLKVYKIPEASRIAMGKAIVNLISLAP-<br>DEKIMATLSTKDFSDERSLAFFTKNGVVKRTNLSEFESNRSCGIRAIVLDEGDELVSAKVVDKNAKHLLIASHLGIFIKFPLEEVREIGRTTRGVIGIKLNENDFVVGAVVISDDGNKLLSVSENGLGKQTLA<br>EAYRGQSRGGKGVIGMKLTQKTGNLVGVISVDDENLDLMILTASAKMIRVSIKDIRETGRNASGVKLINTADKVMYVNSCPKEEEPENLETSSAQNLFE

Sa<br>mp<br>le<br>04	KPVHRRILYAMHELGLTSKVAYKKSARIVGDVIGK-<br>YHPHGDTAVYDALVRMAQDFSMRLELVDGQGNFGSIDGDNAAAMRYTEARMTKASEEILRDIDKDTIDFVPNYDDTLKEPDILPSRLPNLLVNGANGIAVGMATSIPPHRIDEIIDALA<br>HVLENPNAELDEILEFV	N87T,<br>M191I,<br>V199A,<br>G208E
Sa<br>mp<br>le<br>06	KPVHRRILYAMHELGLTSKVAYKKSARIVGDVIGKYHPHGDI-<br>AVYDALVRMAQDFSMRLELVDGQGNFGSIDGDNAAAMRYTEARMTKASEEILRDIDKDTIDFVPNYDDTLKEPDILPSRLPNLLVNGANGIAVGMATSIPPHRIDEIIDALAHVLENPN<br>AELDEILEF	N87I,<br>M191I,<br>V199A,<br>G208E
Sa<br>mp<br>le<br>07	LKPVHRRILYAMHELGLTSKVAYKKSARIVGDVIGKYHPHGDNA-<br>VYDALVRMAQDFSMRLELVDGQGNFGSIDGDNAAAMRYTEARMTKASEEILRDIDKDTIDFVPNYDDTLKEPDILPSRLPNLLVNGANGIAVGMATSIPPHRIDEIIDALAHVLENPNA<br>ELDEI	M191I,<br>V199A,<br>G208E
Sa<br>mp<br>le<br>09	VHRRILYAMHELGLTSKVAYKKSARIVGDVIGKYHPHGDNA-<br>VYDALVRMAQDFSMRLELVDGQGNFGSIDGDNAAAMRYTEARMTKASEEILRDIDKETIDFVPNYDDTLKEPDILPSRLPNLLVNGANGIAVGMATSIPPHRIDEIIDALVHVLENPNAE<br>LDEI	D145E,<br>M191I,<br>G208E
Sa<br>mp	LKPVHRRILYAMHELGLTSKVAYKKSARIVGDVIGKYHPHGDNA-<br>VYDALVRMAQDFSMRLELVDGQGNFGSIDGDNAAAMRYTEARMTKASEEILRDIDKDTIDFVPNYDDTLKEPDILPSRLPNLLVNGANGIAVGMATSIPPHRIDEIIDALVHVLENPNAE<br>LDEI	M191I,<br>G208E

le 11		
Sample 13	LKPVHRRILYAMHELGLTSKVAYKKSARIVGDVIGKYHPHGDNA-<br>VYDALVRMAQDFSMRLELVDGQGNFGSIDGDNAAAMRYTEARMTKASEEILRDIDKDTIDFVPNYDDTLKEPDILPSRLPNLLVNGANGIAVGMATSIPPHRIDEIIDALAHVLENPNA<br>ELDEILEFVKGA	M191I, V199A, G208E, P219A
Sample 14	LKPVHRRILYAMHELGLTSKVAYKKSARIVGDVIGKYHPHGDNA-<br>VYDALVRMAQDFSMRLELVDGQGNFGSIDGDNAAAMRYTEARMTKASEEILRDIDKDTIDFVPNYDDTLKEPDILPSRLPNLLVNGANGIAVGMATSIPPHRIDEIIDALAHVLENPNA<br>ELDEILEFV	M191I, V199A, G208E
Sample 15	HRRILYAMHELGLTSKVAYKKSARIVGDVIGKYHPHGDNA-<br>VYYALVRMAQDFSMRLELVDGQGNFGSIDGDNAAAMRYTEARMTKASEEILRDIDKDTIDFVPNYDDTLKEPDILPSRLPNLLVNGANGIAVGMATSIPPHRIDEIIDALAHVLENPNAE<br>LDEILEFVK	D91Y, M191I, V199A, G208E
Sample 17	VHRRILYAMHELGLTSKVAYKKSARIVGDVIGKYHPHGDNA-<br>VYDALVRMAQDFSMRLELVDGQGNFGSIDGDNAAAMRYTEARMTKASEEILRDIDKDTIDFVPNYDDTLKEPDILPSRLPNLLVNGANGIAVGMATSIPPHRMDEIIDALAHVLENP	V199A
Sample 20	LPDARDGLKPVHRRILYAMHELGLTSKVAYKKSARIVGDVIGK-<br>YHPHGDKAVYDALVRMAQDFSMRLELVDGQGNFGSIDGDNAAAMRYTEARMTKASEEILRDIDKDTIDFVPNYDDTLKEPDILPSRLPNLLVNGANGIAVGMATSIPPHRIDEIIDALA<br>HYL	N87K, M191I, V199A, V201Y
Sample 22	LKPVHRRILYAMHELGLTSKVAYKKSARIVGDVIGKYHPHGDNA-<br>VYDALVRMAQDFSMRLELVDGQGNFGSIDGDNAAAMRYTEARMTKASEEILRDIDKDTIDFVPNYDDTLKEPDILPSRLPNLLVNGANGIAVGMATSIPPHRIDEIIDALVHVLENPNAE<br>LDEILEFVK	M191I, G208E
Sample 23	LKPVHRRILYAMHELGLTSKVAYKKSARIVGDVIGKYHPHGDNA-<br>VYDALVRMAQDFSMRLELVDGQGNFGSIDGDNAAAMRYTEARMTKASEEILRDIDKDTIDFVPNYDDTLKEPDILPSRLPNLLVNGANGIAVGMATSIPPHRIDEIIDALVHVLENPNAE<br>LDEILEF	M191I, G208E
Sample 24	LKPVHRRILYAMHELGLTSKVAYKKSARIVGDVIGKYHPHGDNA-<br>VYDALVRMAQDFSMRLELVDGQGNFGSIDGDNAAAMRYTEARMTKASEEILRDIDKDTIDFVPNYDDTLKEPDILPSRLPNLLVNGANGIAVGMATSIPPHRIDEIIDALAHVLENPNA<br>ELDEILEFV	M191I, V199A, G208E
Sample 25	LKPVHRRILYAMHELGLTSKVAYKKSARIVGDVIGKYHPHGDNA-<br>VYDALVRMAQDFSMRLELVDGQGNFGSIDGDNAAAMRYTEARMTKASEEILRDIDKDTIDFVPNYDDTLKEPDILPSRLPNLLVNGANGIAVGMATSIPPHRIDEIIDALVHVLENPNAE<br>LDEILEFVKGPD	M191I, G208E
Sample 28	LKPVHRRILYAMHELGLTSKVAYKKSARIVGDVIGKYHPHGDNA-<br>VYDALVRMAQDFSMRLELVDGQGNFGSIDGDNAAAMRYTEARMTKASEEILRDIDKDTIDFVPNYDDTLKEPDILPSRLPNLLVNGANGIAVGMATSIPPHRIDEIIDALVHVLENPNAE<br>LDEILEFVKGPD	M191I, G208E

Table S3. Sequence of amino acids from our reference RdxA and the samples studied.

WP_000670110.1	MKFLDQEKRRQLLNE**R**H**S**C**K**MFDSHY-<br>EFSSTELEEIAEIARLSP**SSY**NTQPWHFVMVTDKDLKKQIAAHSYF**N**EEMIKSASALMVVCSLRPSELLPHGHYMQNLYPESYKVRVIPSFAQMLGVRFNHSMQRLESYILE<br>QCYIAVGQICMGVSLMGLDSC**IIGG**FDPLKVGEVLEERINKPKIACLIALGKRVAEASQ**K**S**R**KSKVDAITWL

Q6H	MKFLD**H**EKRRQLLNERHSCKMFDSHY-<br>EFSSTELEEIAEIARLSPSSYNTQPWHFVMVTDKDLKKQIAAHSYFNEEMIKSASALMVVCSLRPSELLPHGHYMQNLYPESYKVRVIPSFAQ<br>MLGVRFNHSMQRLESYILEQCYIAVGQICMGVSLMGLDSCIIGGFDPLKVGEVLEERINKPKIACLIALGKRVAEASQKSRKSKVDAITWL	---
R16H	MKFLDQEKRRQLLNE**H**HSCKMFDSHY-<br>EFSSTELEEIAEIARLSPSSYNTQPWHFVMVTDKDLKKQIAAHSYFNEEMIKSASALMVVCSLRPSELLPHGHYMQNLYPESYKVRVIPSFAQ<br>MLGVRFNHSMQRLESYILEQCYIAVGQICMGVSLMGLDSCIIGGFDPLKVGEVLEERINKPKIACLIALGKRVAEASQKSRKSKVDAITWL	Known to reduce ox-<br>idonuclease affinity
S30R	MKFLDQEKRRQLLNERHSCKMFDSHYEFS**R**-<br>TELEEIAEIARLSPSSYNTQPWHFVMVTDKDLKKQIAAHSYFNEEMIKSASALMVVCSLRPSELLPHGHYMQNLYPESYKVRVIPSFAQMLG<br>VRFNHSMQRLESYILEQCYIAVGQICMGVSLMGLDSCIIGGFDPLKVGEVLEERINKPKIACLIALGKRVAEASQKSRKSKVDAITWL	---
M56V	MKFLDQEKRRQLLNERHSCKMFDSHY-<br>EFSSTELEEIAEIARLSPSSYNTQPWHFV**V**VTDKDLKKQIAAHSYFNEEMIKSASALMVVCSLRPSELLPHGHYMQNLYPESYKVRVIPSFAQ<br>MLGVRFNHSMQRLESYILEQCYIAVGQICMGVSLMGLDSCIIGGFDPLKVGEVLEERINKPKIACLIALGKRVAEASQKSRKSKVDAITWL	Known to destabilise<br>dimer formation
D59N	MKFLDQEKRRQLLNERHSCKMFDSHY-<br>EFSSTELEEIAEIARLSPSSYNTQPWHFVMVT**N**KDLKKQIAAHSYFNEEMIKSASALMVVCSLRPSELLPHGHYMQNLYPESYKVRVIPSFAQ<br>MLGVRFNHSMQRLESYILEQCYIAVGQICMGVSLMGLDSCIIGGFDPLKVGEVLEERINKPKIACLIALGKRVAEASQKSRKSKVDAITWL	High frequency rate
R90K	MKFLDQEKRRQLLNERHSCKMFDSHY-<br>EFSSTELEEIAEIARLSPSSYNTQPWHFVMVTDKDLKKQIAAHSYFNEEMIKSASALMVVCSL**K**PSELLPHGHYMQNLYPESYKVRVIPSFAQ<br>MLGVRFNHSMQRLESYILEQCYIAVGQICMGVSLMGLDSCIIGGFDPLKVGEVLEERINKPKIACLIALGKRVAEASQKSRKSKVDAITWL	High frequency rate
H97T	MKFLDQEKRRQLLNERHSCKMFDSHY-<br>EFSSTELEEIAEIARLSPSSYNTQPWHFVMVTDKDLKKQIAAHSYFNEEMIKSASALMVVCSLRPSELLP**T**GHYMQNLYPESYKVRVIPSFAQ<br>MLGVRFNHSMQRLESYILEQCYIAVGQICMGVSLMGLDSCIIGGFDPLKVGEVLEERINKPKIACLIALGKRVAEASQKSRKSKVDAITWL	---
G98S	MKFLDQEKRRQLLNERHSCKMFDSHY-<br>EFSSTELEEIAEIARLSPSSYNTQPWHFVMVTDKDLKKQIAAHSYFNEEMIKSASALMVVCSLRPSELLPH**S**HYMQNLYPESYKVRVIPSFAQ<br>MLGVRFNHSMQRLESYILEQCYIAVGQICMGVSLMGLDSCIIGGFDPLKVGEVLEERINKPKIACLIALGKRVAEASQKSRKSKVDAITWL	High frequency rate
P106L	MKFLDQEKRRQLLNERHSCKMFDSHY-<br>EFSSTELEEIAEIARLSPSSYNTQPWHFVMVTDKDLKKQIAAHSYFNEEMIKSASALMVVCSLRPSELLPHGHYMQNLY**L**ESYKVRVIPSFAQ<br>MLGVRFNHSMQRLESYILEQCYIAVGQICMGVSLMGLDSCIIGGFDPLKVGEVLEERINKPKIACLIALGKRVAEASQKSRKSKVDAITWL	Known to cause loss of<br>function of the reduc-<br>tase
S108A	MKFLDQEKRRQLLNERHSCKMFDSHY-<br>EFSSTELEEIAEIARLSPSSYNTQPWHFVMVTDKDLKKQIAAHSYFNEEMIKSASALMVVCSLRPSELLPHGHYMQNLYPE**A**YKVRVIPSFAQ<br>MLGVRFNHSMQRLESYILEQCYIAVGQICMGVSLMGLDSCIIGGFDPLKVGEVLEERINKPKIACLIALGKRVAEASQKSRKSKVDAITWL	---
A118T	MKFLDQEKRRQLLNERHSCKMFDSHY-<br>EFSSTELEEIAEIARLSPSSYNTQPWHFVMVTDKDLKKQIAAHSYFNEEMIKSASALMVVCSLRPSELLPHGHYMQNLYPESYKVRVIPSF**T**Q<br>MLGVRFNHSMQRLESYILEQCYIAVGQICMGVSLMGLDSCIIGGFDPLKVGEVLEERINKPKIACLIALGKRVAEASQKSRKSKVDAITWL	High frequency rate

V123T	MKFLDQEKRRQLLNERHSCKMFDSHY-EFSSTELEEIAEIARLSPSSYNTQPWHFVMVTDKDLKKQIAAHSYFNEEMIKSASALMVVCSLRPSELLPHGHYMQNLYPESYKVRVIPSFAQMLG**T**RFNHSMQRLESYILEQCYIAVGQICMGVSLMGLDSCIIGGFDPLKVGEVLEERINKPKIACLIALGKRVAEASQKSRKSKVDAITWL	New mutation
R131K	MKFLDQEKRRQLLNERHSCKMFDSHY-EFSSTELEEIAEIARLSPSSYNTQPWHFVMVTDKDLKKQIAAHSYFNEEMIKSASALMVVCSLRPSELLPHGHYMQNLYPESYKVRVIPSFAQMLGVRFNHSMQ**K**LESYILEQCYIAVGQICMGVSLMGLDSCIIGGFDPLKVGEVLEERINKPKIACLIALGKRVAEASQKSRKSKVDAITWL	High frequency rate
V172I	MKFLDQEKRRQLLNERHSCKMFDSHY-EFSSTELEEIAEIARLSPSSYNTQPWHFVMVTDKDLKKQIAAHSYFNEEMIKSASALMVVCSLRPSELLPHGHYMQNLYPESYKVRVIPSFAQMLGVRFNHSMQRLESYILEQCYIAVGQICMGVSLMGLDSCIIGGFDPLKVGE**I**LEERINKPKIACLIALGKRVAEASQKSRKSKVDAITWL	---
A183V	MKFLDQEKRRQLLNERHSCKMFDSHY-EFSSTELEEIAEIARLSPSSYNTQPWHFVMVTDKDLKKQIAAHSYFNEEMIKSASALMVVCSLRPSELLPHGHYMQNLYPESYKVRVIPSFAQMLGVRFNHSMQRLESYILEQCYIAVGQICMGVSLMGLDSCIIGGFDPLKVGEVLEERINKPKI**V**CLIALGKRVAEASQKSRKSKVDAITWL	Known to reduce ox-idonuclease affinity

Binding residues mentioned by Martínez-Júlvez *et al.* [4] are formatted in bold and coloured green. Mutations from our samples in the sequences are formatted in bold and coloured red. Italics were used to signal samples with mutations in positions known to cause some effect. Samples with new mutations are underlined

```
    Score        Expect     Identities       Gaps       Strand
311 bits(168)    2.00E-88 306/372(82%)    12/372(3%)   Plus/Plus

E. coli         1773  ACTGTTTATTAAAAACACAGCACTGTGCAAACACGAAAGTGGACGTATACGGTGTGACGC  1832
                      ||||||||  |||||||||||||| ||| ||| || ||| ||| ||||| ||||||||||
HPrrnA23S_PCR   1859  ACTGTTTACCAAAAACACAGCACTTTGCCAACTCGTAAGAGGAAGTATAAGGTGTGACGC  1918

E. coli         1833  CTGCCCGGTGCCGGAAGGTTAA-TTGATGGGGTTAG-CGCAAG-CGAAGCTCTTG-ATCG  1888
                      |||||||||||  |||||||||   ||| | || || ||||||  |||||  ||| || |
HPrrnA23S_PCR   1920  CTGCCCGGTGCTCGAAGGTTAAGAGGAT-GCGTCAGTCGCAAGATGAAGC-GTTGAATTG  1976

E. coli         1889  AAGCCCCGGTAAACGGCGGCCGTAACTATAACGGTCCTAAGGTAGCGAAATTCCTTGTCG  1948
                      ||||||  ||||||||||||||||||||||||||||||||||||||||||||||||||||
HPrrnA23S_PCR   1977  AAGCCCGAGTAAACGGCGGCCGTAACTATAACGGTCCTAAGGTAGCGAAATTCCTTGTCG  2036

E. coli         1949  GGTAAGTTCCGACCTGCACGAATGGCGTAATGA--TGGCCAGGCTGTCTCCACCCGAGAC  2006
                      | ||| | |||||||||| ||||||||||| ||  |||  | |||||||| ||| |||| 
HPrrnA23S_PCR   2037  GTTAAATACCGACCTGCATGAATGGCGTAACGAGATGGG-A-GCTGTCTCAACCAGAGAT  2094

E. coli         2007  TCAGTGAAATTGAACTCGCTGTGAAGATGCAGTGTACCCGCGGCAAGACGGAAAGACCCC  2066
                      |||||||||||| | | |  ||||| || |    ||||||||||||||||||||||||||
HPrrnA23S_PCR   2095  TCAGTGAAATTGTAGTGGAGGTGAAAATTCCTCCTACCCGCGGCAAGACGGAAAGACCCC  2154

E. coli         2067  GTGAACCTTTACTATAGCTTGACACTGAACATTGAGCCT-TGATGTGTAGGATAGGTGGG  2125
                      ||| |||||||||| | |||  |||||   || | |  | | ||| | ||||||||||||
HPrrnA23S_PCR   2155  GTGGACCTTTACTACAACTTAGCACTGCTAATGG-GAATATCATGCGCAGGATAGGTGGG  2213

E. coli         2126  AGGCTTTGAAGT  2137
                      ||||||||||||
HPrrnA23S_PCR   2214  AGGCTTTGAAGT  2225
```

Figure S1. Alignment between 23S rRNA from E. coli and the PCR amplification of HPrrnA23S.

```
SP|P0AES4|GYRA_ECOLI NDWNKAYKKSARVVGDVIGKYHPHGDSAVYDTIVRMAQPFSLRYMLVDGQGNFGSIDGDS 116
SP|P48370|GYRA_HELPY LTSKVAYKKSARIVGDVIGKYHPHGDNAVYDALVRMAQDFSMRLELVDGQGNFGSIDGDN 120
                        : *******:*************.****::***** **:*  **************.

SP|P0AES4|GYRA_ECOLI AAAMRYTEIRLAKIAHELMADLEKETVDFVDNYDGTEKIPDVMPTKIPNLLVNGSSGIAV 176
SP|P48370|GYRA_HELPY AAAMRYTEARMTKASEEILRDIDKDTIDFVPNYDDTLKEPDILPSRLPNLLVNGANGIAV 180
                     ******** *::* :.*:: *::*:*:*** ***.* * **::*:::*******:.****
```

Figure S2. Region QRDR of the alignment of E. coli K12 (P0AES4) and H. pylori 26695 (P48370). QRDR regions are coloured red (71-73) and purple (74-110). CAP domains are coloured purple (74-110) and blue (110-145).

Moxifloxacin

```
sp|P48370|GYRA_HELPY        LGLTSKVAYKKSARIVGDVIGKYHPHGDNAVYDALVRMAQDFSMRLELVDGQGNFGSIDG 118
5BS8:A|PDBID|CHAIN|SEQUENCE SGFRPDRSHAKSARSVAETMGNYHPHGDASIYDSLVRMAQPWSLRYPLVDGQGNFGSPGN 120
5BTA:A|PDBID|CHAIN|SEQUENCE SGFRPDRSHAKSARSVAETMGNYHPHGDSSIYDSLVRMAQPWSLRYPLVDGQGNFGSPGN 120
4Z2C:A|PDBID|CHAIN|SEQUENCE LGVTPDKPHKKSARITGDVMGKYHPHGDSSIYEAMVRMAQWWSYRYMLVDGHGNFGSMDG 112
5CDQ:A|PDBID|CHAIN|SEQUENCE QGMTPDKSYKKSARIVGDVMGKYHPHGDSSIYEAMVRMAQDFSYRYPLVDGQGNFGSMDG 106
                             *.  .  : **** ..:.:*:****** ::*:::***** :* *  ****:***** ..

sp|P48370|GYRA_HELPY        DNAAAMRYTEARMTKASEEILRDIDKDTIDFVPNYDDTLKEPDILPSRLPNLLVNGANGI 178
5BS8:A|PDBID|CHAIN|SEQUENCE DPPAAMRYTEARLTPLAMEMLREIDEETVDFIPNYDGRVQEPTVLPSRFPNLLANGSGGI 180
5BTA:A|PDBID|CHAIN|SEQUENCE DPPAAMRYTEARLTPLAMEMLREIDEETVDFIPNYDGRVQEPTVLPSRFPNLLANGSGGI 180
4Z2C:A|PDBID|CHAIN|SEQUENCE DSAAAQRYTEARMSKIALEMLRDINKNTVDFVDNYDANEREPLVLPARFPNLLVNGATGI 172
5CDQ:A|PDBID|CHAIN|SEQUENCE DGAAAMRYTEARMTKITLELLRDINKDTIDFIDNYDGNEREPSVLPARFPNLLANGASGI 166
                            *  ** ******::  : *:**:*:::*:**: ***   :** :**:*:****.**: **
```

Levofloxacin

```
sp|P48370|GYRA_HELPY        ELGLTSKVAYKKSARIVGDVIGKYHPHGDNAVYDALVRMAQDFSMRLELVDGQGNFGSID 117
5BTG:A|PDBID|CHAIN|SEQUENCE DSGFRPDRSHAKSARSVAETMGNYHPHGDASIYDSLVRMAQPWSLRYPLVDGQGNFGSPG 119
5BTI:A|PDBID|CHAIN|SEQUENCE DSGFRPDRSHAKSARSVAETMGNYHPHGDSSIYDSLVRMAQPWSLRYPLVDGQGNFGSPG 119
4Z2D:A|PDBID|CHAIN|SEQUENCE ELGVTPDKPHKKSARITGDVMGKYHPHGDSSIYEAMVRMAQWWSYRYMLVDGHGNFGSMD 111
                            : *.  .  : **** ..:.:*:****** ::*:::***** :* *  ****:***** .

sp|P48370|GYRA_HELPY        GDNAAAMRYTEARMTKASEEILRDIDKDTIDFVPNYDDTLKEPDILPSRLPNLLVNGANG 177
5BTG:A|PDBID|CHAIN|SEQUENCE NDPPAAMRYTEARLTPLAMEMLREIDEETVDFIPNYDGRVQEPTVLPSRFPNLLANGSGG 179
5BTI:A|PDBID|CHAIN|SEQUENCE NDPPAAMRYTEARLTPLAMEMLREIDEETVDFIPNYDGRVQEPTVLPSRFPNLLANGSGG 179
4Z2D:A|PDBID|CHAIN|SEQUENCE GDSAAAQRYTEARMSKIALEMLRDINKNTVDFVDNYDANEREPLVLPARFPNLLVNGATG 171
                            .*  ** ******::  : *:**:*:::*:**: ***   :** :**:*:****.**: *
```

Trovafloxacin

```
sp|P48370|GYRA_HELPY        LTSKVAYKKSARIVGDVIGKYHPHGDNAVYDALVRMAQDFSMRLELVDGQGNFGSIDGDN 120
4Z2E:A|PDBID|CHAIN|SEQUENCE VTPDKPHKKSARITGDVMGKYHPHGDSSIYEAMVRMAQWWSYRYMLVDGHGNFGSMDGDS 114
                            :* .  :******.***:********.::*:*:***** :* *  ****:*****:***.
```

Ciprofloxacin

```
sp|P48370|GYRA_HELPY        DGLKPVHRRILYAMHELGLTSKVAYKKSARIVGDVIGKYHPHGDNAVYDALVRMAQDFSM 102
5BTC:A|PDBID|CHAIN|SEQUENCE DGLKPVHRRVLYAMFDSGFRPDRSHAKSARSVAETMGNYHPHGDSSIYDSLVRMAQPWSL 104
2XCT:B|PDBID|CHAIN|SEQUENCE DGLKPVHRRILYGLNEQGMTPDKSYKKSARIVGDVMGKYHPHGDSSIYEAMVRMAQDFSY 300
                            *********:**.: : *:  . :: **** *.:.:*:******.::*:::***** :* 

sp|P48370|GYRA_HELPY        RLELVDGQGNFGSIDGDNAAAMRYTEARMTKASEEILRDIDKDTIDFVPNYDDTLKEPDI 162
5BTC:A|PDBID|CHAIN|SEQUENCE RYPLVDGQGNFGSPGNDPPAAMRYTEARLTPLAMEMLREIDEETVDFIPNYDGRVQEPTV 164
2XCT:B|PDBID|CHAIN|SEQUENCE RYPLVDGQGNFGSMDGDGAAAMRFTEARMTKITLELLRDINKDTIDFIDNYDGNEREPSV 360
                            *  ********** ..*  ****:****:*  : *:**:*:::*:**: ***.  :** :
```

Gatifloxacin

```
SP|P48370|GYRA_HELPY        LGLTSKVAYKKSARIVGDVIGKYHPHGDNAVYDALVRMAQDFSMRLELVDGQGNFGSIDG 118
5BTD:A|PDBID|CHAIN|SEQUENCE SGFRPDRSHAKSARSVAETMGNYHPHGDASIYDSLVRMAQPWSLRYPLVDGQGNFGSPGN 120
5BTF:A|PDBID|CHAIN|SEQUENCE SGFRPDRSHAKSARSVAETMGNYHPHGDSSIYDSLVRMAQPWSLRYPLVDGQGNFGSPGN 120
                             *:  . :: **** *.:.:*:****** ::**:****** :*:*  ********** ..

SP|P48370|GYRA_HELPY        DNAAAMRYTEARMTKASEEILRDIDKDTIDFVPNYDDTLKEPDILPSRLPNLLVNGANGI 178
5BTD:A|PDBID|CHAIN|SEQUENCE DPPAAMRYTEARLTPLAMEMLREIDEETVDFIPNYDGRVQEPTVLPSRFPNLLANGSGGI 180
5BTF:A|PDBID|CHAIN|SEQUENCE DPPAAMRYTEARLTPLAMEMLREIDEETVDFIPNYDGRVQEPTVLPSRFPNLLANGSGGI 180
                            *  *********:*  : *:**:**::*:**:****. ::** :****:****.**:.**
```

Figure S3. Alignment of P48370 to other structures complexed with fluoroquinolones. Positions with mutations on samples are marked in red. DNA GyrA interactions with fluoroquinolones are coloured as follows: blue for non-bonded contacts, green for H-bonds, and purple for both. Amino acids underlined are mutations present in the original structure.
